# Enhancing patient safety by integrating ethical dimensions to Critical Incident Reporting Systems

**DOI:** 10.1186/s12910-021-00593-8

**Published:** 2021-03-08

**Authors:** Kai Wehkamp, Eva Kuhn, Rainer Petzina, Alena Buyx, Annette Rogge

**Affiliations:** 1 Department of Internal Medicine I, University Hospital Schleswig-Holstein, Campus Kiel, Arnold-Heller Str. 3 (K3), 24105 Kiel, Germany; 2 Department of Medical Management, MSH Medical School Hamburg, Hamburg, Germany; 3 Section Global Health, Institute of Hygiene and Public Health, University Hospital Bonn, Bonn, Germany; 4 Quality and Risk Management, Patient Safety, University Hospital Schleswig-Holstein, Kiel, Germany; 5 Department of Human Medicine, MSH Medical School Hamburg, Hamburg, Germany; 6 Institute of History and Ethics in Medicine, Technical University of Munich, Munich, Germany; 7 Department of Medical Ethics, University Hospital Schleswig-Holstein, Kiel, Germany

**Keywords:** Ethics management, CIRS, Risk management, Quality management, Categorization, Healthcare

## Abstract

**Background:**

Critical Incident Reporting Systems (CIRS) provide a well-proven method to identify clinical risks in hospitals. All professions can report critical incidents anonymously, low-threshold, and without sanctions. Reported cases are processed to preventive measures that improve patient and staff safety. Clinical ethics consultations offer support for ethical conflicts but are dependent on the interaction with staff and management to be effective. The aim of this study was to investigate the rationale of integrating an ethical focus into CIRS.

**Methods:**

A six-step approach combined the analysis of CIRS databases, potential cases, literature on clinical and organizational ethics, cases from ethics consultations, and experts’ experience to construct a framework for CIRS cases with ethical relevance and map the categories with principles of biomedical ethics.

**Results:**

Four main categories of critical incidents with ethical relevance were derived: (1) patient-related communication; (2) consent, autonomy, and patient interest; (3) conflicting economic and medical interests; (4) staff communication and corporate culture. Each category was refined with different subcategories and mapped with case examples and exemplary related ethical principles to demonstrate ethical relevance.

**Conclusion:**

The developed framework for CIRS cases with its ethical dimensions demonstrates the relevance of integrating ethics into the concept of risk-, quality-, and organizational management. It may also support clinical ethics consultations’ presence and effectiveness. The proposed enhancement could contribute to hospitals’ ethical infrastructure and may increase ethical behavior, patient safety, and employee satisfaction.

## Background

Health care professionals act in complex settings that are challenging with a diversity of structures, processes, and relations. To provide a high standard of medical care, they have to apply a wide variety of continuously updated knowledge and skills. However, even if health professionals intend to act carefully and responsibly, there is an almost constant risk of unintendedly harm patients. Therefore, patient safety improvement became an increasingly important goal in most healthcare systems [1]. Within this context, risk management (RM) is a methodological framework to systematically identify and assess potential risks and threats to prevent and minimize potential hazards [2]. Quality management (QM) is a complementary bundle of methodologies to increase the quality of structures, processes, and assorted outcomes [3, 4]. QM and RM have substantial overlaps within the organizational setting and are often combined in an integrated management system (quality- and risk management—QRM). One of the commonly used methods to deal with risks in the context of healthcare-related RM and QM is the Critical Incident Reporting System (CIRS) [5, 6]: CIRS is a reporting system for critical clinical incidents and near misses and is set either for internal organizational use (e.g., hospitals) or as a public platform. It provides anonymous, low-threshold access to reporting issues that employees might feel uncomfortable disclosing personally [7]. Both medical and non-medical staff can give input. Patient safety experts assess each case, integrate experts, and deduce suggestions to prevent recurrence and improve patient, staff, and organizational safety. To be successful, a CIRS has to guarantee blame-free, strictly confidential reporting and an independent analysis by experts that give constructive feedback to the incident. To obtain sustainable effects, resulting measures should be compulsory constituted in the QRM system [8–10].

Usually, reported incidents are classical clinical problems, e.g., medication mix-up (look-alike/sound-alike medication) and a subsequent measure, e.g., the introduction of patient identification bracelets. However, hazards to patients may also include ethical dimensions. Examples of this can be found either in the context of clinical ethics, e.g., a report of a disputable practice in end-of-life care, or in organizational ethics, e.g., a critical shortage of staff resulting in increased patient mortality [11, 12].

### Research goal

Ethics are a relevant aspect of quality, and hence, the risk of unethical behavior is also a quality risk. Therefore, we postulated that it might be valuable to enhance the classic RM tool CIRS by explicitly adding an ethical dimension. A classic CIRS supports the improvement of organizational management and clinical quality. An ethically enhanced CIRS might foster the improvement of organizational ethics and clinical ethics. Both may contribute to the concept of ethics management, an organizational framework to support ethical behavior in hospitals and other institutions [13, 14].

The objective of this study was to analyze the ethical dimensions of both real and possible critical incidents to develop a set of categories for potential ethical CIRS cases. These categories could help to argue for a consideration of ethical dimensions within CIRS and thereby contribute to patient safety and the development of an ethics management framework.

##  Methods

A six-step approach combining empirical and theoretical elements was used to compile a comprehensive framework of categories for potential CIRS cases with ethical dimensions. It is based on the evaluation of authentic and possible critical incidents. The method for analysis and categorization is based on Qualitative Content Analysis [15]. The six steps that were performed included: (1) Anonymous CIRS cases from January 2018 to March 2019 with ethical aspects reported at a German hospital were identified and analyzed. The selection criterion was the attribute “ethical case”, which could be selected either actively by the reporting hospital personnel or by the analyzing CIRS staff. (2) Cases of clinical ethics counseling within the same period and hospital were analyzed concerning organizational ethics, organizational risks, and management. (3) Authentic CIRS cases from three openly accessible, inter-institutional, German-speaking CIRS databases were analyzed systematically regarding related ethical aspects [“CIRS medical”, “CIRS Health Care”, “Jeder Fehler zählt” (“Every error counts”)^1^]. (4) The resulting examples were complemented with other possible cases, based on a literature search and the author's clinical, organizational, and ethical experiences. The literature search focused on the related context of patient safety, clinical and organizational ethics. (5) The collection of identified authentic and possible cases was mapped and analyzed to deduce abstracted issues/subcategories and generalized main categories for probable ethical CIRS cases. In terms of Qualitative Content Analysis, these main categories are an abstracting reduction of the cases eligible for coding. Each subcategory was complemented with a typical case example. (6) To fathom and demonstrate ethical relevance, each subcategory was complemented with the primary affected Principles of Biomedical Ethics (i.e., respect for autonomy, non-maleficence, beneficence, justice) [16]. The categories were not weighted or sequenced. As a cross-check for feasibility, real cases were assigned to the framework.

## Results

The evaluation of ethical cases from the openly available CIRS databases, cases from internal hospital CIRS, and ethics consultation combined with the literature search and the authors’ experience resulted in a comprehensive set of possible ethical CIRS cases. Four main categories for ethical critical incidents/cases were derived: (1) patient-related communication; (2) consent, autonomy, and patient interest; (3) conflicting economic and medical interests; (4) staff communication and corporate culture. The subsidiary subcategories that specify potential issues, the exemplary cases, and the potentially related ethical principles are shown in Tables 1, 2, 3 and 4. Cross-check with real cases showed that an assignment to suitable categories is feasible, although their multiple facets and complexity sometimes overlap between different categories or subcategories. The primarily relevant biomedical principles could be mapped roughly to demonstrate ethical relevance, but the cross-check showed that the ensemble of relevant principles often has to be adapted in practice.

**Table 1:** Categories of ethical CIRS cases: patient-related communication (focus on staff and patients)

Potential issue/subcategory	Example	Potentially related ethical principles
Patient communication of errors or incidents	A patient suffers from hypoxic brain injury due to suboptimal resuscitation conducted by insufficiently trained medical staff. The cause of the complication is communicated neither to the patient nor her relatives	Autonomy Non-maleficence Beneficence
Communication of bad news	A physician exaggerates and sugarcoats an end-stage lung cancer patient's prognosis to reduce his stress of breaking bad news	Autonomy Non-maleficence Beneficence
Professional/respectful communication	A physician informs a patient about a severe diagnosis on a crowded hospital corridor	Non-maleficence Beneficence

**Table 2:** Categories of ethical CIRS cases: consent, autonomy, and patient interest (focus on staff and patients)

Potential issue/subcategory	Example	Potentially related ethical principles
Patient participation and shared decision making	A chief physician orders his staff to softly dismiss patient's questions on risks and alternative options before elective surgery	Autonomy Non-maleficence Beneficence
Impaired/reduced consciousness; incapacity to give consent	At an intensive-care ward, a septic patient in artificial coma is getting dialysis and several antibiotic therapy cycles, although her patient's provision states a limitation of life-prolonging measures	Autonomy Non-maleficence Beneficence
Conflicts between well-being and autonomy; physical restraint	Patients at a geriatric ward are regularly put under physical and medical restraint to reduce nursing staff's care requirements	Autonomy Non-maleficence Beneficence
Withhold/withdraw treatment; end of life decisions; assisted dying; advance healthcare directives	A physician puts pressure on the wife of a patient with severe Alzheimer's disease to consent to a feeding tube, although she argues it would not be in his interest	Autonomy Non-maleficence Beneficence
Intercultural conflicts	A Muslima denies a gynecologic surgery that would be performed by a male physician but does not dare to name the reasons	Autonomy Non-maleficence

**Table 3:** Categories of ethical CIRS cases: conflicting economic and medical interests (focus on organization, staff and patients)

Potential issue/subcategory	Example	Potentially related ethical principles
Economic incentives on medical decisions	The management of a hospital pays a bonus to senior physicians if they perform enough surgeries. Several patients have spine surgery because of lower back pain without preceding alternative conservative options	Non-maleficence Justice
Allocation decisions, spatial and staff resources	The management of a hospital puts the scarce staff resources to the department with the best financial return of investment. Other departments are understaffed, although they have to provide care for patients with even higher needs	Justice Beneficence

**Table 4:** Categories of ethical CIRS cases: staff communication and corporate culture (focus on staff and organization)

Potential issue/subcategory	Example	Potentially related ethical principles
Team communication of errors or incidents	Decisions that interns made during night shifts are convicted hard and unfair by a senior physician the next morning. As a consequence, only the non-ambiguous cases are presented. Difficult decisions are delayed	Beneficence Non-maleficence
Critical incident stress debriefing; second victim	A resident has confused two medications and does not dare to disclose the error because she works in a climate of fear. Consequently, the patient suffers from mysterious symptoms (side effects of the wrong medication) and gets many futile diagnostics	Beneficence Non-maleficence
Conflicting interdisciplinary medical objectives	Interdisciplinary decisions in a hospital are delayed because of personal conflicts between the surgeon and internal medicine specialists	Beneficence Non-maleficence

Tables 1, 2, 3 and 4*Categories of ethical CIRS-cases* with primarily involved groups (focus), examples, and exemplary related ethical principles. Patients regularly include relatives or other patient-related persons. Possibly related ethical principles are simplified to demonstrate the ethical relevance, notwithstanding that in practice, the ethical discussion is often extensively complex.

## Discussion

The qualitative analysis of ethical aspects of CIRS cases shows the variety and significance of possible critical incidents that originate in insufficiently handled ethical conflicts. It demonstrates the importance of the ethical dimension within the clinical and the organizational setting and supports the case to extend the scope of risk and quality management by adding an ethical perspective to CIRS.

### A framework for critical ethical incidents

The proposed categories are a first attempt to map possible ethical CIRS cases in the clinical context. It may provide risk managers with a helpful framework to analyze reported cases and to sensitize staff for respective reporting. For this purpose, the framework is simplified, and real-life cases may routinely match more than one category or subcategory. The assigned principles of biomedical ethics are also generalized and simplified. They are not meant to cover the complexity of real-life issues but to demonstrate the ethical impact of potential CIRS cases. Therefore, the discussion of ethical conflicts in real CIRS cases underlies the same challenge as other structured reflections on clinical and organizational ethics. The exemplary stated principles have to be complemented with an appropriate individual ethical argumentation that may consider other principles, values, or concepts [17–20].

#### Patient-related communication

The first category addresses communication problems between hospital staff and patients or their relatives. Reported incidents range from unprofessional or disrespectful communication to deficits in the communication of errors or bad news, all connected to frequently occurring situations [21–24]. A communication deficit can reduce the patient's autonomy and cause direct harm [25, 26]. On the other hand, good communication can contribute to the patient's well-being—either directly or by improving the patient’s possibilities [27, 28]. Sometimes, the staff also suffers as the recipient of communication or other interaction with patients, often raising conflicts between employees’ safety and the patient’s autonomy and the right to medical treatment. Such incidents are often accompanied by a lack of resources and training (associated with category 3) and neglected aftercare (associated with category 4) [29].

#### Consent, autonomy, and patient interest

The second category includes issues that are common conflicts of clinical ethics like considerations between the autonomy and well-being of patients, assisted dying or withholding or withdrawing of therapy, shared decision making, and others. While autonomy, beneficence, and non-maleficence may be the prominently discussed principles, arguments on distributive or restorative justice may be indicated in other constellations. CIRS cases in this context appear if the case-immanent conflict is not solved properly, leaving patients or members of staff with ethical doubts or moral distress. [30–34]. The conflicts addressed in this category are also quite common in regular clinical ethics consultations [35–37].

#### Conflicting economic and medical interests

The third category reaches from precarious medical decisions that result in economic benefit for staff or management and concurrently potentially harmful consequences for patients to situations where difficult allocation decisions in respect to spatial, staff, or other resources are solved insufficiently. Economic or financial constraints or incentives can lead to a substantial conflict of interest between medical treatment quality and financial profit or costs. At one extreme, treatment decisions are significantly influenced by reimbursement decisions, taking a non-indicated medical treatment into account [38, 39]. At the other extreme, not enough resources are provided, for example, to save expenditures for investment or employees. Consequently, the prioritization of relatively scarce goods may necessary, tackling the ethical principle of distributive justice, beneficence, and non-maleficence [40, 41].

#### Staff communication and corporate culture

The fourth category subsumes problems related to internal communication, medical professionalism, and culture. Insufficient communication between colleagues can lead to decreased patient safety and suboptimal medical treatment [42]. Depending on the actual content, respect for a patient’s autonomy, non-maleficence, or beneficence cannot be preserved. Additionally, also constricted justice or values like honesty or loyalty may be emphasized. [20] Employees’ well-being can be harmed by cultural deficiencies, missing aftercare following stressful events, or second victim situations [43]. Affected medical professionalism is often closely connected to patient-related categories but may also appear inherently and cause critical ethical situations that may be reported via CIRS [44, 45].

It is difficult to distinguish between CIRS cases with and without an ethical dimension. CIRS report suboptimal situations and conditions in patient care, and therefore every incident is potentially harming patients, staff, or society. Frequently reported examples are violations of protocols, i.e., not processed standard-operating procedures, missing checklists, or avoidable treatment errors [46, 47]. The bottom line is that medical treatment and ethics are intrinsically tied to each other: avoidable flawed processes, reduced quality, inadequate medical care are always unethical, and vice versa, sufficient ethical considerations are an immanent aspect of good quality. Following this interpretation, every CIRS case has an ethical dimension that cannot be denied. However, in “not ethical” CIRS cases, there is no dispute on ethical principles. For instance, if a patient is harmed because a physician mixes up the surgery side, the error is indisputably a violation of the do-not-harm principle—there is no need for an ethical discussion. If the same error is not communicated adequately, there may be a conflict between the patient’s autonomy, beneficence, non-maleficence on the one hand, and the physician’s or organization’s interests on the other hand. For this work, we focused on the latter type of cases, therefore CIRS cases with a possible underlying unsolved ethical conflict and a potential increase of moral distress among staff or patients [48]. Of course, in practice, boundaries between ethical and not-ethical incidents are fluid, and precise discrimination is neither always possible nor important: the goal is not a unique assignment as an “either…or” but an augmentation in the sense of a “not only…but also” and thus practically emphasizing the ethical reflection and problem-solving.

It may also occur that cases are reported referring to situations with a deliberate assault on a patient (associated with category 2), bribery (associated with category 3), sexual harassment, or mobbing (associated with category 4). In theory, these cases would fit into the beforementioned categories, but in practice, respective cases are not in the domain of a CIRS and should be assigned to a criminal investigation.^2^ However, if cases with a potential criminal dimension appear in CIRS, the handling is difficult because CIRS ought to be anonymous and blame-free [49]. Definitely, with their relatedness to whistleblowing systems, CIRS offer a low-threshold medium to report these kinds of cases, imposing other interesting ethical questions [50].

### Organizational integration and ethics infrastructure

The standard procedure to process regular CIRS cases within hospitals is a stepwise approach: staff has the opportunity to report critical cases anonymously (usually via an online form) and optionally to attribute characteristics of the underlying problem (like “interdisciplinary” or “communication”). A risk manager renders the case anonymous, prepares it for analysis, and assigns it to related stakeholders or specialists to further evaluate and deduce possible measures (e.g., asking a senior surgeon for comment; advice to implement a safety checklist). For in-depth analysis of critical clinical incidents, the London Protocol provides a broadly consented framework. It emphasizes the management’s responsibility to affect the structures and processes regarding seven main factors that continuously underly possible incidents: institutional, organizational, work environment, team, individual, task-related, and patient factors [49, 51]. Eventually, the cases and derived measures are often presented in an internal database to support crowd learning [49].

While the common focus of CIRS is based on the medical and organizational perspective of risk and quality management, emphasizing additional ethical perspectives seems important. Therefore, we suggest that attention should be paid to CIRS cases with a disputable ethical issue. Employees and risk managers should be trained to sense critical ethical incidents. The cases should be presented to an internal clinical ethics committee or specialists for clinical ethical consultations [52]. These ethic experts may push for analysis and discussions on the appropriate levels to solve the specific case, foster referring ethical competency, and prevent similar problems.

Therefore, the formal process of the ethical attribution of CIRS cases lowers clinicians' threshold to address corresponding cases and supports a link between quality- and risk management and ethical instances like an ethics committee or ethics consultants [53].

The ethical instances should be integrated on an appropriate organizational level to aim for effective ethics management, tightly connected to quality and risk management. The concern for clinical and organizational issues that underly ethical conflicts may contribute to a better ethical and organizational culture and increase the quality of care [54–57].

The explicit amendment of optional ethical attributes for CIRS cases, proper handling with ethical expertise, and integration into organizational management contributes to an ethical infrastructure’s formal elements. Additionally, the commitment to ethics management also affects the critical informal and cultural ethical development. It is important to keep in mind that introducing a formal ethical infrastructure (like the ethical attribution of CIRS cases) without proper handling of the resulting cases also poses a risk to increase unethical behavior. In the worst case, half-hearted management measures may foster a cultural feeling that ethics are just a superficial label without real importance and unethical practice is without consequences [58].

### Limitations

This study aimed to analyze and discuss possible critical ethical incidents that may be a relevant subject of an ethically augmented CIRS. The framework and categories that were derived are a first suggestion. It is not based on a representative sample but mostly on confidential and publicly available CIRS databases, a focused literature analysis, and the authors’ personal clinical, organizational, and ethical experiences. It is important to state that—ultimately—each framework is a construction that has to prove its feasibility in practice. Therefore, the framework is not meant to rigorously categorize real incidents but foster awareness and consideration of critical ethical incidents in general. Especially the exemplary illustrated ethical principles are not universally applicable. Depending on the actual case constellation and the related ethical or cultural system, other or additional values, beliefs, or ethical reasonings have to be considered.

## Conclusion

The four categories, *patient-related communication*, *patient interest*, *conflicting interest*, and *corporate culture* address potential ethical conflicts in healthcare organizations with a need for ethics consultation. Additional empirical research is needed to investigate the ethical enhanced CIRS’s influence on patient safety, ethical behavior, and organizational culture. We would welcome a further discussion of the proposed framework and the ethical impact. Despite the limitations stated above, this article illustrates the rationale, feasibility, and potentially positive implications of considering an ethical perspective within CIRS. The integration of CIRS and a comprehensive ethics management framework with an effective bond to organizational, risk, and quality management may enhance patient safety, ethical behavior, and corporate culture in hospitals and other healthcare providers.

## Data Availability

Original CIRS data of hospitals is subject to high privacy and not disclosed. Data of public CIRS databases used for this work can be achieved online (www.cirsmedical.de, www.cirs-health-care.de, www.jeder-fehler-zaehlt.de).

## Abbreviations

QM: Quality management
QRM: Quality and risk management
RM: Risk management
CIRS: Critical Incident Reporting System(s)

## Footnotes

1 www.cirsmedical.de; www.cirs-health-care.de; www.jeder-fehler-zaehlt.de

2 Some organizations assign this domain to a “compliance officer”.

## References

[1] Kohn LT, Corrigan JM, Donaldson MS (2000). To err is human: building a safer health system.

[2] Briner M, Kessler O, Pfeiffer Y, Wehner T, Manser T (2010). Assessing hospitals' clinical risk management: development of a monitoring instrument. BMC Health Serv Res.

[3] Øvretveit J (2000). Total quality management in European healthcare. Int J Health Care Qual Assur.

[4] Takeda H, Matsumura Y, Nakajima K, Kuwata S, Zhenjun Y, Shanmai J (2003). Health care quality management by means of an incident report system and an electronic patient record system. Int J Med Inform.

[5] Blehle S (2014). Risikomanagement und Fehlervermeidung im Krankenhaus.

[6] Osterloh F. Qualitätssicherung: Zehn Jahre Fehlermeldesysteme. Dtsch Arztebl. 2019;115:A-715/B-587/C-575.

[7] Vrbnjak D, Denieffe S, O'Gorman C, Pajnkihar M (2016). Barriers to reporting medication errors and near misses among nurses: a systematic review. Int J Nurs Stud.

[8] Khorsandi M, Skouras C, Beatson K, Alijani A (2012). Quality review of an adverse incident reporting system and root cause analysis of serious adverse surgical incidents in a teaching hospital of Scotland. Patient Saf Surg.

[9] Mahajan RP (2010). Critical incident reporting and learning. Br J Anaesth.

[10] Petschnig W, Haslinger-Baumann E (2017). Critical Incident Reporting System (CIRS): a fundamental component of risk management in health care systems to enhance patient safety. Saf Health.

[11] Griffiths P, Maruotti A, Recio Saucedo A, Redfern OC, Ball JE, Briggs J, et al. Nurse staffing, nursing assistants and hospital mortality: retrospective longitudinal cohort study. BMJ Qual Saf; 2018:bmjqs-2018-008043. 10.1136/bmjqs-2018-008043 PMC6716358 30514780

[12] Ravenscroft AJ, Bell MD (2000). "End-of-life" decision making within intensive care–objective, consistent, defensible?. J Med Ethics.

[13] Snellman CL (2015). Ethics management: how to achieve ethical organizations and management?. Bus Manag Educ.

[14] Wehkamp K, Wehkamp K-H (2017). Ethikmanagement im Krankenhaus : Unternehmens- und Wertekultur als Erfolgsfaktor für das Krankenhaus [Ethics management in hospitals: corporate culture and culture of values as a factor for success].

[15] Schreier M (2012). Qualitative content analysis in practice.

[16] Beauchamp TL, Childress JF (2001). Principles of biomedical ethics.

[17] Friedrich O, Hemmerling K, Kuehlmeyer K, Nörtemann S, Fischer M, Marckmann G (2017). Principle-based structured case discussions: do they foster moral competence in medical students? A pilot study. BMC Med Ethics.

[18] Banerjee D, Kuschner WG (2007). Principles and procedures of medical ethics case consultation. Br J Hosp Med.

[19] Steinkamp N, Gordijn B (2003). Ethical case deliberation on the ward. A comparison of four methods. Med Health Care Philos.

[20] Christen M, Ineichen C, Tanner C (2014). How, "moral" are the principles of biomedical ethics?—A cross-domain evaluation of the common morality hypothesis. BMC Med Ethics.

[21] Makary MA, Daniel M (2016). Medical error-the third leading cause of death in the US. BMJ.

[22] Munjal S (2017). Breaking bad news. Curr Psychiatr.

[23] Gallagher TH, Studdert D, Levinson W (2007). Disclosing harmful medical errors to patients. N Engl J Med.

[24] Tigard DW (2019). Taking the blame: appropriate responses to medical error. J Med Ethics.

[25] Ubel PA, Scherr KA, Fagerlin A (2017). Empowerment failure: how shortcomings in physician communication unwittingly undermine patient autonomy. Am J Bioethics.

[26] Bell SK, White AA, Yi JC, Yi-Frazier JP, Gallagher TH (2017). Transparency when things go wrong: physician attitudes about reporting medical errors to patients, peers, and institutions. J Patient Saf.

[27] Jotterand F, Amodio A, Elger BS (2016). Patient education as empowerment and self-rebiasing. Med Health Care Philos.

[28] Onguti S, Mathew S, Todd C (2018). Communication and ethics in the clinical examination. Med Clin N Am.

[29] Schablon A, Zeh A, Wendeler D, Peters C, Wohlert C, Harling M (2012). Frequency and consequences of violence and aggression towards employees in the German healthcare and welfare system: a cross-sectional study. BMJ Open.

[30] Bringedal B, Isaksson Rø K, Magelssen M, Førde R, Aasland OG. Between professional values, social regulations and patient preferences: medical doctors' perceptions of ethical dilemmas. J Med Ethics; 2017:medethics-2017-104408. 10.1136/medethics-2017-104408 29151056

[31] Berger ZD, Boss EF, Beach MC (2017). Communication behaviors and patient autonomy in hospital care: a qualitative study. Patient Educ Couns.

[32] Mentzelopoulos SD, Slowther AM, Fritz Z, Sandroni C, Xanthos T, Callaway C (2018). Ethical challenges in resuscitation. Intensive Care Med.

[33] Lemiengre J, de Casterlé BD, Van Craen K, Schotsmans P, Gastmans C (2007). Institutional ethics policies on medical end-of-life decisions: a literature review. Health Policy.

[34] Tan J (2003). The anorexia talking?. Lancet.

[35] DuVal G, Sartorius L, Clarridge B, Gensler G, Danis M (2001). What triggers request for ethics consultations?. J Med Ethics.

[36] Hurst SA, Perrier A, Pegoraro R, Reiter-Theil S, Forde R, Slowther AM (2007). Ethical difficulties in clinical practice: experiences of European doctors. J Med Ethics.

[37] Saarni SI, Halila R, Palmu P, Vänskä J (2008). Ethically problematic treatment decisions in different medical specialties. J Med Ethics.

[38] Stachura P, Oberender P, Bundscherer AC, Wiese CHR (2015). The possible impact of the German DRGs reimbursement system on end-of-life decision making in a surgical intensive care unit. Wien Klin Wochenschr.

[39] Biller-Andorno N, Lenk C, Leititis J (2004). Ethics, EBM, and hospital management. J Med Ethics.

[40] Schürmann J, Meyer-Zehnder B, Mertz M, Schleger HA, Schlögl M, Kressig RW, Pargger H, Reiter-Theil S, Danis M, Hurst SA, Fleck LM, Førde R, Slowther A (2015). Fairness and transparency in bedside micro-allocation. Fair resource allocation and rationing at the bedside.

[41] Schäfer J, Hensen P, Kölzer C (2011). Ressourcenallokation im Krankenhaus – Akteure zwischen Medizin und Ökonomie. Die gesunde Gesellschaft. Sozioökonomische Perspektiven und sozialethische Herausforderungen.

[42] Hohenstein C, Hempel D, Schultheis K, Lotter O, Fleischmann T (2014). Critical incident reporting in emergency medicine: results of the prehospital reports. Emerg Med J.

[43] Miller L (1999). Critical incident stress debriefing: clinical applications and new directions. Int J Emerg Ment Health.

[44] DuBois JM, Anderson EE, Chibnall JT, Mozersky J, Walsh HA (2019). Serious ethical violations in medicine: a statistical and ethical analysis of 280 cases in the United States from 2008–2016. Am J Bioeth.

[45] Buyx AM, Maxwell B, Schöne-Seifert B (2008). Challenges of educating for medical professionalism: who should step up to the line?. Med Educ.

[46] Shillito J, Arfanis K, Smith A (2010). Checking in healthcare safety: theoretical basis and practical application. Int J Health Care Qual Assur.

[47] Hales BM, Pronovost PJ (2006). The checklist—a tool for error management and performance improvement. J Crit Care.

[48] Lamiani G, Borghi L, Argentero P (2017). When healthcare professionals cannot do the right thing: a systematic review of moral distress and its correlates. J Health Psychol.

[49] Howell AM, Burns EM, Hull L, Mayer E, Sevdalis N, Darzi A (2017). International recommendations for national patient safety incident reporting systems: an expert Delphi consensus-building process. BMJ Qual Saf.

[50] Blenkinsopp J, Snowden N, Mannion R, Powell M, Davies H, Millar R (2019). Whistleblowing over patient safety and care quality: a review of the literature. J Health Organ Manag.

[51] Vincent C, Taylor-Adams S, Chapman EJ, Hewett D, Prior S, Strange P (2000). How to investigate and analyse clinical incidents: clinical risk unit and association of litigation and risk management protocol. BMJ.

[52] McLean SAM (2007). What and who are clinical ethics committees for?. J Med Ethics.

[53] Orlowski JP, Hein S, Christensen JA, Meinke R, Sincich T (2006). Why doctors use or do not use ethics consultation. J Med Ethics.

[54] Opel DJ, Brownstein D, Diekema DS, Wilfond BS, Pearlman RA (2009). Integrating ethics and patient safety: the role of clinical ethics consultants in quality improvement. J Clin Ethics.

[55] Sine DM, Sharpe VA (2011). Ethics, risk, and patient-centered care: how collaboration between clinical ethicists and risk management leads to respectful patient care. J Healthc Risk Manag.

[56] Nelson WA, Gardent PB, Shulman E, Splaine ME (2010). Preventing ethics conflicts and improving healthcare quality through system redesign. Qual Saf Health Care.

[57] Opel DJ, Wilfond BS, Brownstein D, Diekema DS, Pearlman RA (2009). Characterisation of organisational issues in paediatric clinical ethics consultation: a qualitative study. J Med Ethics.

[58] Tenbrunsel AE, Smith-Crowe K, Umphress EE (2003). Building houses on rocks: the role of the ethical infrastructure in organizations. Soc Justice Res.

